# Random-Reaction-Seed Method for Automated Identification of Neurite Elongation and Branching

**DOI:** 10.1038/s41598-019-39962-0

**Published:** 2019-02-27

**Authors:** Alvason Zhenhua Li, Lawrence Corey, Jia Zhu

**Affiliations:** 1 Vaccine and Infectious Disease Division, Fred Hutchinson Cancer Research Center, Seattle, WA 98109 USA; 2 Clinical Research Division, Fred Hutchinson Cancer Research Center, Seattle, WA 98109 USA; 3 Department of Laboratory Medicine, University of Washington, Seattle, WA 98195 USA; 4 Department of Medicine, University of Washington, Seattle, WA 98195 USA

## Abstract

Conventional deterministic algorithms (i.e., skeletonization and edge-detection) lack robustness and sensitivity to reliably detect the neurite elongation and branching of low signal-to-noise-ratio microscopy images. Neurite outgrowth experiments produce an enormous number of images that require automated measurement; however, the tracking of neurites is easily lost in the automated process due to the intrinsic variability of neurites (either axon or dendrite) under stimuli. We have developed a stochastic random-reaction-seed (RRS) method to identify neurite elongation and branching accurately and automatically. The random-seeding algorithm of RRS is based on the hidden-Markov-model (HMM) to offer a robust enough way for tracing arbitrary neurite structures, while the reaction-seeding algorithm of RRS secures the efficiency of random seeding. It is noteworthy that RRS is capable of tracing a whole neurite branch by only one initial seed, so that RRS is proficient at quantifying extensive amounts of neurite outgrowth images with noisy background in microfluidic devices of biomedical engineering fields. The method also showed notable performance for reconstructing of net-like structures, and thus is expected to be proficient for biomedical feature extractions in a wide range of applications, such as retinal vessel segmentation and cell membrane profiling in spurious-edge-tissues.

## Introduction

Neurons undergo the most complicated morphogenesis of all cells in a developing organism. The neuronal developing process (termed as neurite) results in the formation of a complex neuronal architecture where it can be difficult to distinguish between axons and dendrites^1^. Neurite outgrowth is involved in a wide range of extracellular and intracellular stimuli and illnesses. Understanding neurite outgrowth can improve therapeutics for nervous system disease. For instance, the length of neurite extension has been used to quantify the effect of nerve growth proteins for understanding and treating sensory peripheral neuropathies^2–4^.

**Figure 1:**
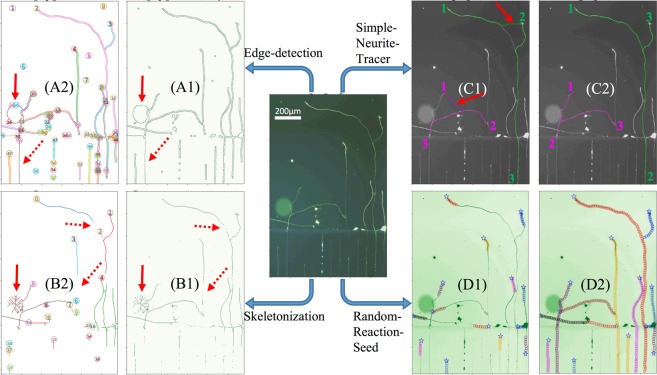
Challenges in analysis of neurite structure in image with low SNR. The central image is the raw neurite image with a phase halo artifact. Both (**A**,**B**) are the results from conventional deterministic algorithms. (**A1**) Resulted edge map from the edge detection method (Canny filter) shows double edges along the borders of the neurite path, and the typical circular structure from halo, as indicated by the solid red arrow marker. This gradient-based method has the problem of missing edge, as indicated by the dashed red arrow marker. (**A2**) A total of 58 sets of connected edge are classified from the edge map (the number within a circle marker is the index of each connected edge). (**B1**) Resulted skeleton map from the skeletonization method^17^ shows disconnected axial line of the neurite path, and the typical radial artifact from halo, as indicated by the solid red arrow marker. This thinning algorithm has the problem of missing/broken skeleton, as indicated by the dashed red arrow marker. (**B2**) A total of 19 sets of connected skeleton is classified from the skeleton map (the number within a circle marker is the index of each connected skeleton). (**C**) The results from the popular semi-manual Simple-Neurite-Tracer (deterministic algorithm + human intelligence). (**C1**) Bad resultant paths from the wrong order of manual-selected successive points (as indicated by the color numbers 1, 2, 3). Two demos are presented, each demo contains 3 successive points with the similar color of the detected paths, in which the wrong path is indicated by the red arrow marker. (**C2**) Good resultant from the right order of manual-selected successive points. These accurate finding paths in each demo are obtained simply by switching the location of “2” and “3” points. (**D**) The results from the Random-Reaction-seed algorithm (RRS). (**D1**) A total of 14 initial seeds, as indicated by the blue pentagon-star, are placed on the tip or junction of the neurite paths, then the RRS algorithm traces down each neurite path starting from the seed. The beginning resultant traces are indicated by the sequence of color circles. (**D2**) A completed and accurate neurite structure with 14 branches is achieved simply by 14 initial seeds, the resultant traces of the RRS algorithm are indicated by color circles on the raw neurite image (green channel).

However, a precise quantification of neurites, especially of dynamic neurite outgrowth in micro-fluidic devices of the biomedical engineering field, is a challenging task that only cutting-edge image analysis techniques can perform successfully and reliably. Conventional deterministic algorithms including skeletonization^5^ and edge-detection^6^ have difficulty and limitations in determining neuronal structures in noisy images. There are several major issues encountered during skeleton/edge processing of images with low signal-to-noise-ratio (SNR). At the beginning, a time-consuming preparation is unavoidable, because a proper skeleton/edge extracted from an image requires an interplay of sharpness adjustments (e.g., threshold level). This process becomes unproductive for a long-stitched image combined with different exposure sub-images. A more problematic and less acceptable result from the deterministic skeleton/edge algorithm is, that the extracted skeletons/edges are often broken pieces due to low SNR images, as indicated by the red dashed arrows in Fig. 1. Other limitations include the generation of artifact signals and false identification of neuronal structures because skeleton/edge algorithms are very sensitive to phase halo effects, as indicated by the red solid arrows in Fig. 1.

To overcome the limitations, certain stochastic algorithms have been introduced in recent years^7–10^. For instance, hidden-Markov-model (HMM), a subclass of dynamic Bayesian networks^11–14^, is particularly useful in addressing more demanding requirements on the accuracy and robustness of the detection^7^. Despite ongoing algorithm improvements, automated efficient methods for neurite identification in low SNR images is typically challenging due to the intrinsic variability of neurite (either axon or dendrite) under stimuli. In this work, we present a robust and efficient Random-Reaction-Seed method (RRS), which underlies the effectiveness of random seeding that occurs in employing the maximum likelihood method in statistical estimation for probabilistic functions of Markov chain.

## Methods

In this section, we present the experimental nature of the raw images. Then, an automatic random-seed tracing algorithm, and a new highly efficient reaction-seed tracing strategy is presented and discussed in detail.

### Neurons in microfluidic chambers

The neurite growth of human neuroblastoma cells and primary sensory neurons was conducted in a two-chamber microfluidic device (Xona Microfluidic)^4^. These studies evaluated the directional neurite growth of differentiated SY5Y cells in a microfluidic device induced by IL-17c^4^.

### Immunofluorescent staining

Neurites were visualized by staining with a PGP9.5 antibody^4^. The raw images were acquired with an inverted microscope using epi-fluorescence method (Nikon Ti-E system). SNR issues are mainly introduced during specimen preparation. For example, dye-conjugates introduce high intensity fluorescent spots and debris from culture introduces dot-like noise. Additionally, the irregular geometric structure of a microfluidic device will cause blurred or out-of-focus images due to objects from other focus plates.

### HMM-based tracing algorithm starting from a random seed

**Figure 2:**
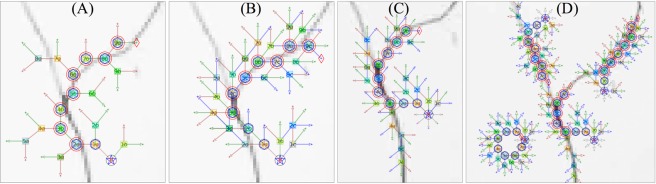
The detail of search process raised by the RRS random seeding. For a clear demonstration of the search process of the RRS method, it is limited to 2 or 3 possible search-paths. The initial seed is denoted as a single blue pentagon-star, the possible path from current-position to predicting-position during each stage is denoted as an arrow-path, and the possible path-destination of a predicting node is denoted as a square-marker. Here, the number inside the marker labels the corresponding node-number while letter (a, b, c) is footnoting the related paths. The maximum-likely path-destination is denoted as a red hexagon-marker. The big red circle-marker indicates a real neurite-object which is evaluated by the local pixel intensity of the predicting node. The interconnection of those big red circle-marker (traced point) realize a continuous neurite tracing. (**A**) In the case of 2 search-paths, one of the simplest cases, the resulted traced points (red circle-marker) has already roughly capturing the actual neurite-object (grey color pixels from actual neurite image). (**B**) In the case of 3 search-paths, the resulted neurite-object identification is more accurate than the case of 2 search-paths. (**C**) The initial search-angle of RRS’s random seed is in the range of 360 degrees, as illustrated by 8 arrow-paths surrounding the pentagon-star, however, for efficiency without losing accuracy, the subsequent search-angles are limited in the range of 90 degrees only. The HMM-based tracing efforts, as indicated in the search arrows, is quite effective to covering all the branches of this junction, but only one branch way is recognized as the neurite path which is indicated by the big red circles. (**D**) Multiple random seeds are needed to trace a branching junction because the efficiency of search performance of a random seed is depending on its starting location. In the case of nothing in the search zone of a seed, the trace of its HMM chain is forming a circular pattern.

RRS is an HMM-based predicting algorithm in which the identification problem is formulated as inferring the discrete hidden variables (position) given observed (pixel-intensity-value). The most likely hidden variable (position) is estimated by maximizing a posterior probability. In order to clearly illustrate the process of neurite-object searching by the RRS random seeding, the pseudocode description is accompanied with a graphical diagram drawing on a real yet simple image (Fig. 2) explaining these essential search steps: (1) computing all possible paths of an HMM-chain, (2) predicting the position of a node of HMM-chain, and (3) identifying an HMM-node as a neurite-object.

First, suppose the HMM chain contains *N* linked events, and each event resulted in a node. Here, the vector $\overrightarrow{v}({A}_{\alpha [n-\mathrm{1]}},{B}_{\beta })$ denotes a forward path starting from a likely present-position *A*_*α*_ of node *N*_*n*−1_ to a next-position *B*_*β*_ with path-length *r* in the image space. For instance, the arrow-path illustrated in Fig. 2 is the corresponding vector $\overrightarrow{v}$. The term ${\mathcal L} ({A}_{\alpha [n-\mathrm{1]}},{B}_{\beta })$ is a sum of the *log* of pixel-intensity-value of all the pixels (linear-interpolation pixels) along the path. The vector $\overrightarrow{V}({A}_{\alpha [n-\mathrm{1]}}^{max},{B}_{\beta [n]})$ denotes a maximum-cost path starting from a present-position ${A}_{\alpha }^{max}$ of *N*_*n*−1_ to a likely next-position *B*_*β*_ of *N*_*n*_. For instance, the squared-marker illustrated in Fig. 2 is the possible path-destination *B*_*β*[*n*]_ of $\overrightarrow{V}$.

**Step 1:**
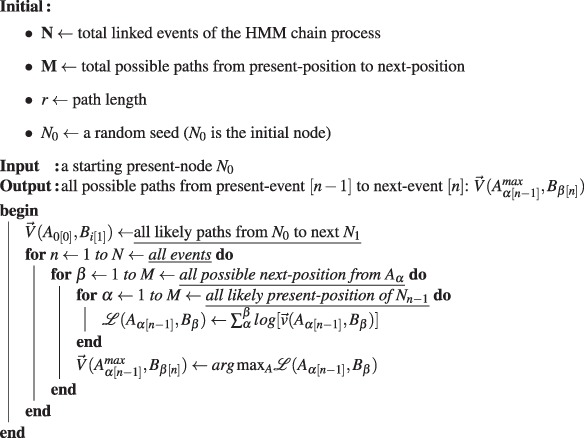
Computing all possible paths to estimate the likely path-destination during each events.

Then, the coming step is looking for the maximum probability path passes through all events, in which the most likely path-destination during each event corresponds to the HMM-node’s physical location *N*_*n*_[*x*, *y*] in the image (*x*, *y* are the coordinates of the pixel where the node is located). This most likely path computation is starting from the final event backtrack to the initial event.

**Step 2:**
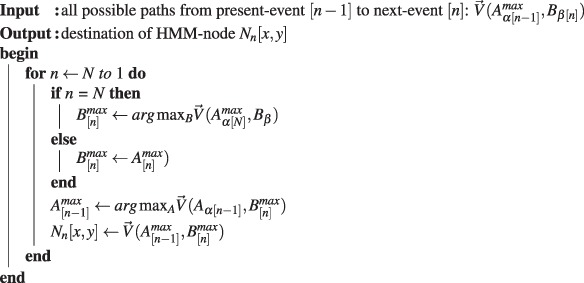
Predicting the HMM-node’s position which is the most likely path-destination during each event.

Here, the vector $\overrightarrow{V}({A}_{[n-\mathrm{1]}}^{max},{B}_{[n]}^{max})$ denotes the maximum-cost path, in which the maximum-likely path-destination is the HMM-node which is denoted as a blue hexagon-marker in Fig. 2. In actual computation during this step, the dynamic algorithm, is applied for speeding up the computation.

Finally, the local area pixel-intensity along the path of two adjacent nodes is applied for checking their connectivity, because the HMM-nodes representing neurite objects are in connection.

**Step 3:**
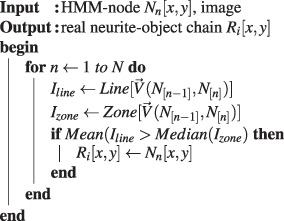
Identifying a HMM-node as a neurite-object by its local pixel-intensity distribution.

Here, the term *I*_*line*_ denotes a one-dimensional pixel-intensity distribution along a vector line from node *N*_[*n*−1]_ to *N*_[*n*]_, while *I*_*zone*_ corresponds to a two-dimensional pixel-intensity distribution of local zone surrounding this path (zone’s width equals to 2 × *line*). The identified neurite-object indicated by a red big-circle-marker in Fig. 2 is evaluated by the local pixel-intensity distribution of the HMM-node. A neurite object *R*_*i*=1_ exists only when the *Median*(*I*_*zone*_) of its surrounding pixels is less than its average pixel-intensity *Mean*(*I*_*line*_). For a successfully recognition of a continuous neurite body, only a chain with at least three neurite-object in sequential order are stored. As indicated in Fig. 2, one seed is limited to identify one piece of neurite branch structure, and sometime the random seed will capture nothing in its searching zone, so that, multiple random seeds are required to cooperate in covering a branching junction.

### Random-reaction-seed strategy

In principle, the minimum required number of initial seeds is equivalent to the total branch number of the neurite. For instance, in a continuous tree-like neurite structure, the proper initial seeds should be located on the tip of each branch as indicated in Fig. 1(D).

However, ideal initial seeds are difficult to generated accurately in advance, because the neurite growth involves arbitrary structures with many faces: scaffold-like, tree-like branching, etc. Adding additional complexity, the neurite object’s morphology in low SNR images is not smoothly continuous, often weakly linked or disturbed by a wide range of artifacts (e.g., optical exposure time, halo-effect-spots, debris). Therefore, the searching algorithm of RRS is designed to apply a randomly distributed seed as an initial seed.

#### Generation of random seed

**Figure 3:**
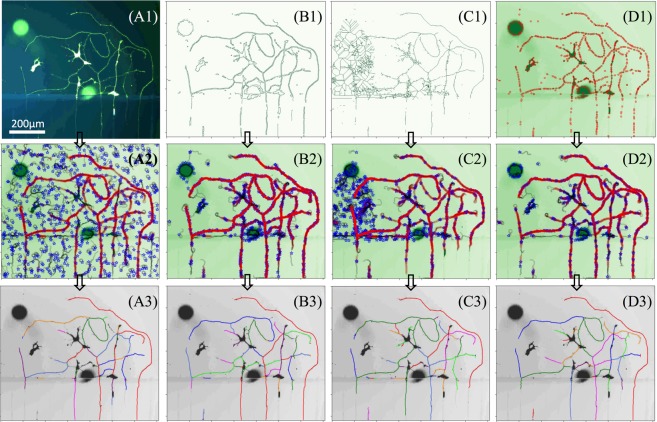
Different approaches of generating random seed distribution. (**A1**) is the raw neurite image with noisy fluorescent spots and artifacts. (**A2**) is the dynamic tracing map from a uniform distribution of random initial seeds which are denoted by blue stars. The active node (i.e., capturing a real neurite object) of a search chain is denoted by a big red-circle while the non-active node is denoted by a small grey-circle. (**A3**) is the traced neurite branches from (**A2**) and colorfully plotted on the raw image (grey-color); (**B1**) is the edge map of (**A1**) by the edge-detection method (Canny filter). (**B2**) is the dynamic tracing on the green-channel image by an edge-like distribution of seeds which are obtained by randomly sampling the edge pixel points in (**B1**). (**B3**) is the traced neurite branches from (**B2**) and colorfully plotted on the raw image (grey-color); (**C1**) is the skeleton map of (A1) by the skeleton-detection method^18^. (**C2**) is the dynamic tracing on the green-channel image by a skeleton-like distribution of seeds which are obtained by randomly sampling the skeleton pixel points in (**C1**). (**C3**) is the traced neurite branches from (**C2**) and colorfully plotted on the raw image (grey-color); (**D1**) is the blob map of (A1) by the blob-detection method (e.g. difference-of-Gaussian). (**D2**) is the dynamic tracing on the green-channel image by a blob-like distribution of seeds which are obtained by randomly sampling the blob pixel points in (**D1**). (**D3**) is the traced neurited branches from (**D2**) and colorfully plotted on the raw image (grey color).

In principle, a uniform distribution of random initial seeds is capable of handling arbitrary neurite structures, as shown in Fig. 3(A2). However, a subtle tracing of locally condensed neurite branches will require a large amount of random seeding to cover a small local detail, while many of the seeds are useless in other non-neurite areas. Therefore, it is desired to obtain an optimal random seed distribution that should be located on the feature points of a neurite structure, so that it is able to achieve faster convergence (i.e., fewer steps for an accurate result).

One simple approach for a quality collection of random seeds is obtained by randomly sampling the skeleton/edge map, which is generated by a conventional skeletonization/edge-detection method. For instance, the random seeds (blue stars) in Fig. 3(C2) are randomly picked up from the skeleton map in Fig. 3(C1). Here, the total number of random seeds for achieving the accurate tracing as illustrated in (C3) is a few percent of the total number of skeleton-pixel-points from the binary pixel map.

Another straightforward approach for a quality collection of random seeds is obtained by randomly sampling the the blob map that is generated by the conventional blob-detection method (e.g., difference-of-Gaussian)^15^. For instance, the random seeds in Fig. 3(D2) are randomly picked up from the blob map in Fig. 3(D1). Here, the total number of random seeds for achieving the accurate tracing as illustrated in (D3) is only a fraction of the total number of blob-points from the blob map.

#### Establishment of reaction seed

**Figure 4:**
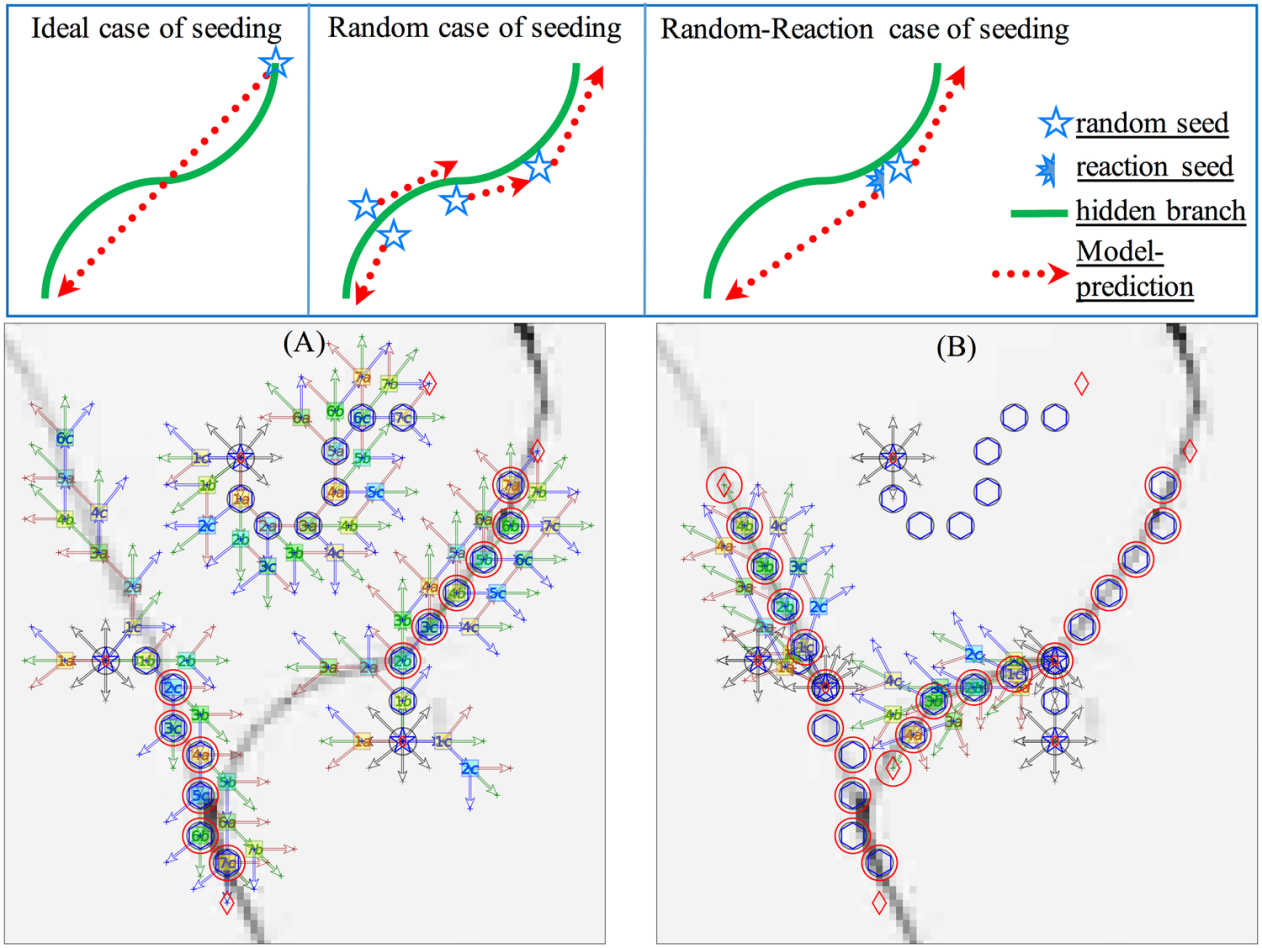
Random-Reaction seeding strategy. The diagrams on the top are illustrating three different cases of seeding, where the red-dotted-arrow denotes the one way prediction (i.e., from present to future) of the HMM-based model. In an ideal case of seeding that a random seed is accidently sitting on the tip of one neurite branch, only one initial seed is required for tracing the whole length of this branch. In the normal random case of seeding, multiple random seeds will be required for covering an entire length of the branch, because one seed will only cover one piece of the branch due to the intrinsic one-way-prediction property of the predictive model. In the Random-Reaction case of seeding, only one active random seed is required to cover the whole length of the branch because its reaction seed offers a guarantee of supplementary tracing. Here, both (**A**,**B**) are the demonstration of random seeds on a neurite image. (**A**) In the normal random case of seeding, a total of 3 random seeds are placed on a neurite image (gray color), accidently, one seed did not trace any neurite while other two seeds are capturing the real neurite objects (i.e., big-red-circle). It is obviously that only part of each branch are covered by the search-chain, so that more seeds are needed to cover this entire junction formed by two branches. (**B**) In the random-reaction case of seeding, the empty circles are the resultant traces from the random seeds of (**A**), while the circles accompanying with search paths in detail are the tracing chain from the reaction seed. It is clearly showing that each reaction seed is derived from the active chain body of a random seed. Noticeably, the initial search-paths of the reaction seed are limited in 180-degree which is quite a contrast to the 360-degree search-paths of the initial random seed.

As indicated in the diagram in Fig. 4, the HMM-based model is a predictive model that has an intrinsic one-way-direction property, meaning that one random seed will be only partially tracing a neurite branch. In general, multiple random seeds are needed to trace a complete branch because the efficiency of search performance of a random seed depends on its starting location. Therefore, it requires a dedicated method to establish a reaction seed for coordinating the completed tracing of a branch.

There are three criteria for the establishment of an efficient reaction seed. (1) The birth condition: a reaction seed is a secondary seed derived from an active chain body of a random seed. Here, an active chain is defined as a search chain that must have at least one node in action to capture a real neurite object. For instance, Fig. 4 depicts the search chain from a random seed with at least one big red circle (e.g., capture a real neurite object) as an active chain. (2) The birth place: a reaction seed is placed on the same location as the first action node (e.g., the first big red circle from the primary chain). (3) The conditional direction: the central search path’s direction should be in the opposite direction of the first two action nodes of the primary chain, while the search-angle range of a reaction seed is better limited to a 180 degree angle, as indicated in Fig. 4(B). Given the above three criteria, the resultant search-chain from this newborn reaction seed will be a supplementary chain to the primary active chain for covering a whole branch of neurite structure.

### HMM-based branching strategy

The outcome from RRS is an interconnected map in binary pixel-value that is ready for further analysis, such as Sholl analysis, radial distance, and total branch length. There are several ways of branching an arbitrary network based on different purposes. Due to the complex construction of stimulated neurites (i.e., lengthy and exaggerated), there is no perfect way to branch apart of a neurite structure. Here, we presented an efficient way of branching an arbitrary network based on the property of RRS that each seed will result in one way tracing. The implementation in a binary pixel net-like image is quite simple. First of all, an initial seed will branch out its own piece, then subsequent seeds will be parting the remaining structure. In a limited recursion, the whole net-like structure will be parting out, as displayed in the Fig. 3(A3,B3,C3,D3).

## Results

In this section, we report comparisons with state-of-the-art algorithms. There is no objective ground truth available for micro-fluidic neurite growth. To assess the accuracy of our method we compare the results with the manual measurement.

### Comparison of RRS with deterministic algorithms

Generally, the accuracy from deterministic algorithms relied heavily on the manual intervention (e.g., human visual intelligence for estimating parameters).

**Figure 5:**
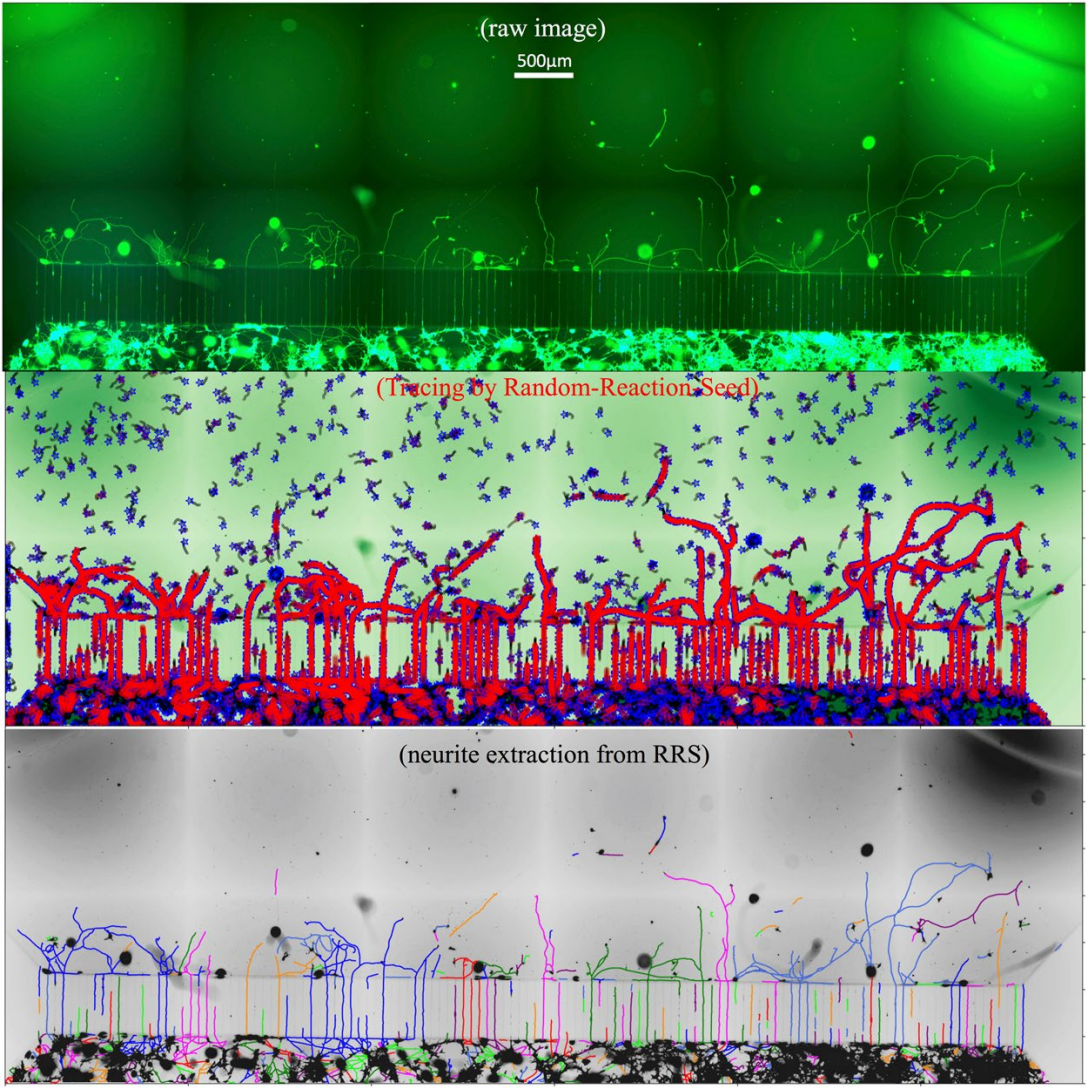
Automated detection of neurite elongation and branching in a stitched image. The image on the top is a typical stitched image with noisy and uneven distribution of fluorescence taken from microfluidic device for neurite development study. The plot in the middle is the dynamic tracing on the raw image (green-channel) by the Random-Reaction-Seed method. The plot on the bottom is the resultant neurite extraction from the above RRS tracing process, and colorfully plotted on the raw image (grey color). Here, each interconnected neurite structure is plotted by one color, so that the connectivity of the entire neurite extraction are observable colorfully.

In the case of applying conventional skeleton/edge-detection deterministic algorithms, the ability to obtain threshold parameters is a prerequisite for performing accurate analysis of an input image. The raw image in Fig. 5 depicts a typical stitched image with noisy and uneven distribution of fluorescence taken from a microfluidic device. It is often a pitfall for applying conventional skeleton/edge-detection, because it is hard to adjust a suitable threshold for the entire long-stitched image: low local contrast will lead to disconnected neurites, while the high local contrast will lead to artifacts from noisy background.

In the case of other deterministic algorithms such as functionals of Hessian matrix, the manual supervision is the key step for accuracy. For instance, Simple Neurite Tracer^16^, a Java-based plug-in module for the popular ImageJ/Fiji^*TM*^, provides an interactive way to recognize a path by using Hessian matrix analysis for curvature evaluation, in which a successful implementation of each neurite branch needs a manual selection process (e.g., manually select the starting point and ending point). This can quickly become a labor-intensive and burdensome task or a impossible if using a large image-set abundant in neurites.

In contrast to the manual intervention of deterministic algorithms, the stochastic RRS algorithm, coded on the open-source Python platform (see Data availability section), is robust against noise and is designed to automatically perform accurate quantification of an extensive number of neuronal images without requiring customization and adjustment of multiple parameters. Furthermore, RRS applies the fast feature generation of input image by deterministic algorithms (i.e., skeleton/edge or blob detection) as the likelihood approach of seeding to enhance the optimization of random seed distribution.

### Comparison of RRS with other HMM-based algorithm

The Neuron Image Analyzer^7^ is a HMM-based tool that relies on soma centroid’s local maxima as the initial point for its neurite tracing. This is particularly useful for neuron images with an outstanding soma cell body, but not for neurite image without specific soma bodies. However, fundamentally, many microfluidic neurite devices are isolated environment so that most soma bodies are compartmented in one chamber while the neurite outgrowth is cultured in another chamber. In addition, the debris in culture media will often be mistakenly recognized as soma bodies. Thus, there is a need for more robust tools for complex neurite images from micro-fluidic devices. Therefore, the RRS method gives considerable effort to focusing on microfluidic neurite outgrowth in which branching structure does not associated with specific soma bodies. Particularly, the most widespread feature describing neurite outgrowth is its total length.

RRS has explicit features that distinguish it from other HMM-based algorithms. (1) Random-seeding algorithm is a robust way to trace the arbitrary net-like structure without specific seeding-point from the structure roots (i.e., soma cell body). (2) Reaction-seeding algorithm is an efficient way to trace a complete branch with only one initial seed. (3) The proposed branching strategy is a unique way to part the complex neurite outgrowth.

Furthermore, besides the universal uniform random seeding, RRS provides various approaches of likelihood-feature random seeding, as shown in Fig. 3. One significant advantage of these seeding approaches is part of the seeds is highly relevant to the neurite profile without high computational cost. On the other hand, the shortcoming of these approaches is that some part of the seeds is just relevant to the noise or neuronal cell body, because these approaches are based on conventional feature detection methods that will either overestimated the neurite profile due to the artifacts or underestimated the neurite profile due to the threshold. However, one superior advantage of the RRS method is the capability to avoid the halo-effect-spot, and the neuronal cell body (somatic body), because the RRS searching algorithm is native to chain-like linear objects (e.g., geometric width is less than the path-length *r*), and is naturally immunized against polygonal objects (e.g., geometric width is bigger than the path-length *r*). Therefore, these seeding approaches required an approximation of the neurite profile estimation so that the implementation of these conventional feature detection methods (e.g., skeleton or blob) are straightforward and fast without adjusting threshold parameters for each image.

### Evaluation of accuracy and efficiency

The field lacks the availability of a ground truth dataset of microfluidic neurite outgrowth that is different with retina vessel or other neuron-based images in which ground truth dataset are publicly available. Simple Neurite Tracer is a tool used by many neural biologists where its improved accuracy is achieved by a combination of human visual intelligence and the optimized Hessian-based parameters. We have accumulated many neurite measurements from Simple Neurite Tracer, which are used for proof-editing the reconstructions obtained by the automated RRS method. This is an efficient and consistent way to verify the accuracy of RRS by comparing the measurements from the Simple Neurite Tracer with neuronal biologist supervision.

**Figure 6:**
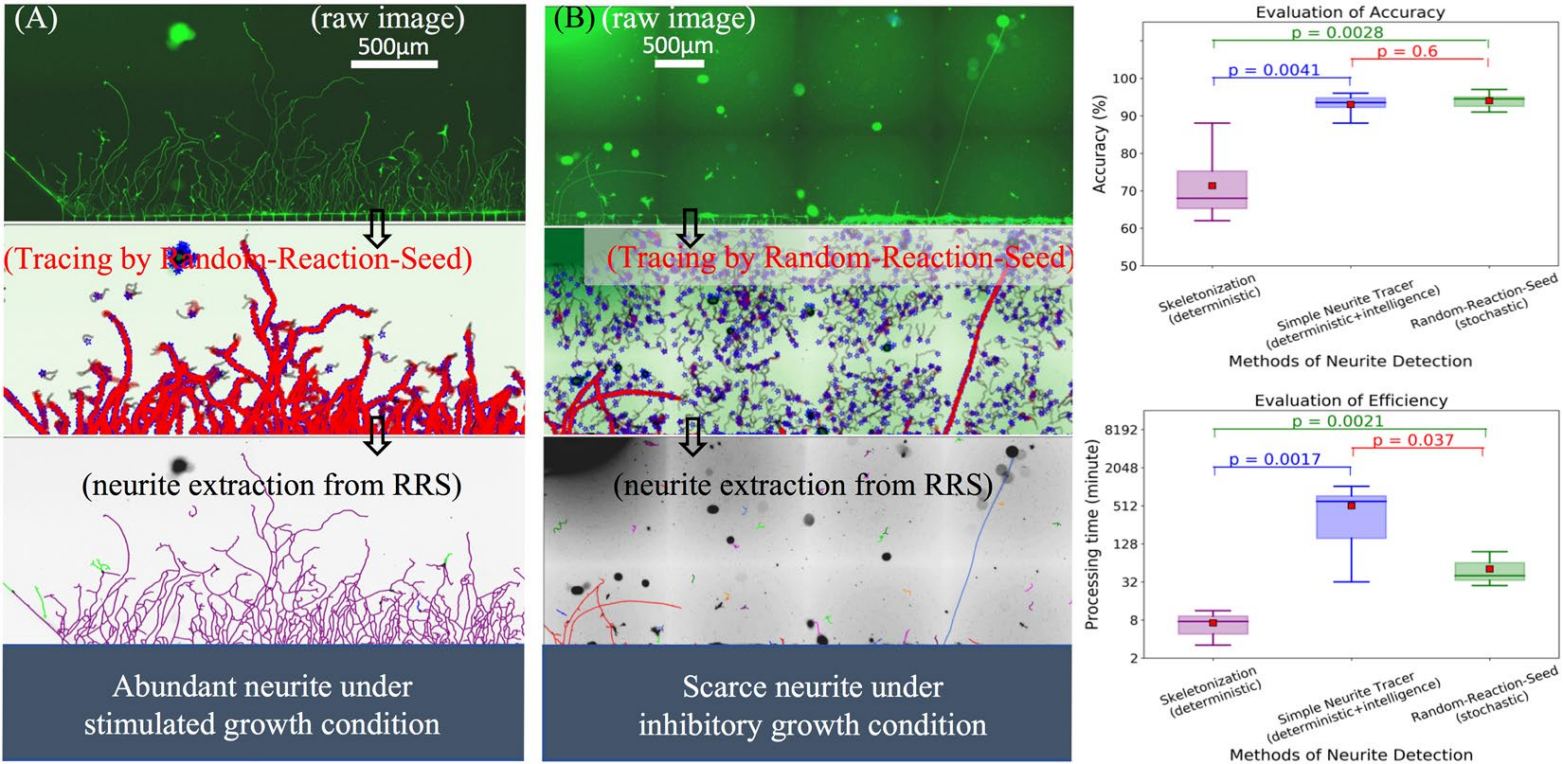
Performance of the RRS method on a diverse imaging dataset under a series of studies evaluating neurite growth^4^. (**A**) In the case of abundant neurite, the processing time between the RRS method and the semi-manual method is close to a 10-fold difference: ~2 hours by RRS, and ~20 hours by Simple Neurite Tracer. (**B**) In the case of scarce neurite, the processing time between the RRS method and the semi-manual method is close to a similar time frame: ~30 minutes by both the RRS and Simple-Neurite-Tracer. Here, on the panels of both (**A**,**B**), the plot on the top is part of a long-stitched raw image, the plot in the middle is the dynamic tracing on the raw image (green channel) by the RRS method, and the plot on the bottom is the resultant neurite extraction from the RRS process, and colorfully plotted on the raw image (grey color). (**C**) The box-plot on the top is the evaluation of accuracy among three different methods, the red-square-point inside each box is the mean value. The accuracies of both the RRS method and Simple-Neurite-Tracer method are significantly higher than the accuracy of the skeleton method (*p* < 0.05), while the accuracy between the RRS method and Simple-Neurite-Tracer method is close to a similar level without significantly different from each other (*p* ≫ 0.05). The box-plot on the bottom is the evaluation of efficiency, the efficiencies of three different methods are significantly different from each other (*p* < 0.05). The p-value is derived from two-sample permutation test.

Here, we present the evaluation of three different methods, as shown in Fig. 6. All the processed images are from our HSV lab, and the computational speed is a processing time per stitched image (e.g., 4096 × 11264 pixels) on a 2.6 GHz Intel Core i7 computer. For evaluating the efficiency, the processing time is a total of exhausted time that includes both the algorithm runtime and consuming time of manual intervention. Importantly, the evaluation of processing time in the semi-manual Simple Neurite Tracer is only a rough estimation, because individual skill and personal patience influences the total amount of exhausting time. For instance, a manual tracing of a high content of neurite branches in a long-stitched image would require days of tedious work per expert, as shown in Fig. 6(A). On the other hand, a manual tracing on a very low content of neurite branches in a long-stitched image would be as productive as the automated RRS method. In the case of scarce neurite growth, the human visual intelligence will take less than a second to recognize a neurite branch, however, the threshold adjustment and parameters selection of the semi-manual tool would take minutes for achieving a better result of a long-stitched image with noisy background, as shown in Fig. 6(B). Specifically, in the semi-manual Simple-Neurite-Tracer with Hessian-based analysis, there are two parameters that can be manually optimized for various noisy neurite structures: Sigma-parameter is the approximate radius of the structures that one can semi-manually trace, and Multiplier parameter is the scaling factor of selecting scale of traced structures. It will then take some time to compute the Gaussian convolution of the image. If it seems to be finding an unexpected path as human visual intelligence or being taking a long time to find an expected path by avoiding noise or artifact, one should cancel the search and try a new start point of the semi-manual tracing. Overall, it will easily take half an hour or more to navigate a long-stitched image with uneven noisy background. Thus, the processing time relies heavily on several facts, including the size of input image, the content/density of neurite branches, and the noisy background.

In order to present an informative evaluation of a great diversity of neurite imaging datasets under a series of studies evaluating neurite growth^4^, we selected six sample images from various neurite growth conditions (stimulated growth, inhibitory growth, and control/normal growth). The representative neurite profile under stimulated growth and inhibitory growth is displayed in Fig. 6, while the representative neurite profile under control/normal growth is similar to the profile in Fig. 5. To determine if two methods are significantly different from each other, the p-value is derived by permutation-test, as this testing is applicable to very small samples.

On average, the skeletonization method (deterministic algorithm) achieves the highest speed but with lowest accuracy, while the RRS (stochastic algorithm) has equivalent accuracy to Simple Neurite Tracer (deterministic & intelligence). Thus, the efficiency of random-reaction-seed method is at least an order of magnitude higher than the semi-manual tool evaluated here. Also, the current CPU-based speed of RRS could be greatly improved by using GPU-based parallelism.

## Discussion

In general, our RRS method is not limited in providing robust object recognition outputs in neurite images taken from microfluidic device, as shown in Fig. 5. It is worth noting that we have started to apply the RRS method on tissue images, in which conventional quantification of nerve fiber growth in skin biopsies relies heavily on manual measurement.

The random-reaction-seed RRS method is an effort to broaden and strengthen the functionality of HMM-based image analysis tools. First of all, beyond the limitations of preset initial points, the random-seeding algorithm has demonstrated the robustness for tracing arbitrary neurite structures. Secondly, the featured reaction-seeding algorithm has demonstrated the efficiency for tracing a completed branch with only one random initial seed. Thirdly, the proposed HMM-based branching strategy provides a systematic way of branching apart of a complex neurite outgrowth. Here, the RRS method has demonstrated the excellent performance in planar image analysis; the RRS method could be easily extended to 3D image analysis. Furthermore, in the case of live-cell imaging with dramatic variation in each sequential imaging frame, this method could potentially offer a high computational accuracy and efficiency to the analysis of live-cell imaging with noisy background. However, in the case of live imaging with small or continuous variation in each sequential imaging frame, it is desired to modify this method to provide better computational efficiency because current implementation of this method is well-suited for static or discontinuous imaging only. For instance, one potentially simple way to modify this method for live-cell imaging would be applying a sequential masking technique to generate the initial seeds efficiently during each sequential imaging processing.

In conclusion, the RRS method has demonstrated the effectiveness and robustness for precise quantification of the length of neurites in large stitched images taken from dynamic and noisy micro-fluidic devices, which is becoming an increasingly useful tool for neural biologists owing to its ability to precisely control, monitor and manipulate neuron-developing micro-environments. Furthermore, the RRS method has the potential applicability and generality on the automated recognition of the growth and branching of peripheral nerves from skin tissue images with various complexities.

Future studies include exploiting the RRS method on the extraction of biomedical elongated structures in a range of applications, such as retinal vessel segmentation and cell membrane profiling in spurious-edge-tissues. A hybrid system combining HMM-based RRS and deep neural learning network will be a promising future investigations.

## Data Availability

The datasets generated and analyzed during the current study are available from the corresponding author on reasonable request. Code for imaging data analysis is available in the repository on GitHub (https://github.com/alvason/identifying_neurite_by_RRS).
